# Trust in nurses and its association with medication adherence of cardiovascular patients: A descriptive correlational study

**DOI:** 10.1016/j.ijnsa.2024.100278

**Published:** 2024-12-05

**Authors:** Ebrahim Aliafsari Mamaghani, Ali Soleimani, Mohammad Zirak

**Affiliations:** Ph.D., Assistant Professor, Research Center for Evidence-Based Health Management, Maragheh University of Medical Sciences, Maragheh, Iran

**Keywords:** Cardiovascular diseases, Patients, Medication adherence, Trust, Nurses, Correlation

## Abstract

**Background:**

Medication adherence plays an important role in managing cardiovascular diseases. Trust in nurses may be effective in enhancing medication adherence in patients with cardiovascular diseases.

**Aim:**

This study aimed to investigate the correlation between trust in nurses among cardiac patients and their medication adherence and explore the predictors of medication adherence.

**Methods:**

This descriptive correlational design was conducted among "302″ cardiac patients hospitalized at Amir Al-momenin Teaching Hospital affiliated with Maragheh University of Medical Sciences. Data was gathered using a demographic characteristics questionnaire, Medication Adherence Scale, and Trust in Nurses’ Scale. Data was analyzed using descriptive (frequency, percentage, mean, standard deviation, median, and descriptive tables) and inferential (Kruskal-Wallis, Mann-Whitney, Spearman correlation coefficient, and quartile regression) statistics. Data was gathered from October 2023 to January 2024. The data was analyzed using SPSS software ver.21.

**Findings:**

The mean and standard deviation of medication adherence and trust in nurses were 91.6 ± 6.9 and 10.5 ± 3.9, respectively. A significant positive correlation was found between trust in nurses and medication adherence(*r* = 0.61). There was a significant difference in medication adherence based on the type of disease. So that, the median medication adherence was the highest for coronary artery patients and the lowest for hypertensive patients. The results of quartile regression analysis showed that trust in nurses and type of disease are the main predictors of medication adherence (R^2^ =20 %.)

**Discussion and conclusions:**

The results indicated that medication adherence among cardiovascular patients was moderate and patients' trust in nurses was less than average. Considering the type of disease as an unmodifiable variable, trust in nurses was the main modifiable factor that plays an important role in increasing medication adherence of cardiovascular patients. Therefore, appropriate strategies such as teaching communication skills to nurses, and training and attracting interested individuals with high communication skills should be taken to enhance patients' trust in nurses.

**What is already known.**

Medication adherence among cardiovascular patients is suboptimal.

Nursing trustworthiness is known to be a predictive factor of medication adherence.

Alt-text: Unlabelled box

**What this paper adds**

The level of patients' trust in nurses is less than moderate.

Cardiac patients have a moderate level of medication adherence.

Trust in nurses positively predicts medication adherence among cardiac patients.

Alt-text: Unlabelled box

## 1 Introduction

Cardiovascular diseases are the main leading cause of death and disability worldwide with an annual of 17 million deaths and 35 million disabilities (Shan et al., 2023). These diseases are among the most prevalent non-communicable diseases in clinical medicine, and the trend of their incidence is increasing due to improved life expectancy, lifestyle changes, sedentary living, increased prevalence of metabolic diseases such as diabetes, environmental pollution, and increased tobacco consumption (Amini et al., 2021). Cardiovascular diseases pose multiple challenges to healthcare systems, families, and cardiac patients. The disruption in individual and social roles, as well as the costs associated with treating and caring for these individuals, are among the most significant issues related to these diseases (Carlsson et al., 2022).

Medication regimens and lifestyle modifications are important components of the treatment protocol for cardiac patients (Simon et al., 2021). Cardiac patients require behavioral interventions and continuous follow-ups to control disease symptoms and prevent disease progression (Simon et al., 2021; Konstantinou et al., 2020). Adherence to a medication regimen is the main key in the management of cardiovascular diseases (Simon et al., 2021; Chew et al., 2019). Patients' adherence means that all patient behaviors should align with the recommendations provided by healthcare providers (Anderson et al., 2020). Adherence to medication leads to better disease control, reduced patient visits to healthcare facilities, and improved quality of life and employment for individuals, ultimately leading to improved disease prognosis. Conversely, non-adherence to treatment leads to recurrent hospitalizations, worsening of prognosis, and death among cardiac patients (Simon et al., 2021; Chen et al., 2022).

It has been found a considerable percentage of cardiac patients do not adhere to their treatment regimens (Anderson et al., 2020; Rashidi et al., 2020; Al-Ganmi et al., 2020). A previous study reported that 51.3 % of patients with coronary artery disease do not adhere to their medical regimen (Zyryanov et al., 2020). Results of a scoping review revealed that medication adherence among patients with hypertension varied from 7 % to 95 % (*M* = 60 %) (Konstantinou et al., 2020). Sajid et al. (2021) reported that the prevalence of non-adherence to antihypertensive medication in Asia was 48 % (Mahmood et al., 2021). Pietrzykowski et al. in a cohort study reported that only 29 % of post-MI patients had sufficient adherence to the medication regimen during the follow-up period. Also, it was found that patients >65 years old and those who had a prior history of coronary artery bypass graft (CABG) mostly had less medication adherence. Whereas, hypertensive and married patients, city inhabitants, and patients with higher education had better medication adherence compared to others (Pietrzykowski et al., 2020). Another study reported that self-efficacy and beliefs about the medication were the main predictors of medication adherence. No significant relationship was found between medication adherence and socio-demographic variables (Al-Ganmi et al., 2020).

Given the importance of medical adherence in the management of cardiovascular diseases- wherein patients must adhere to prescribed treatment for a long duration- this phenomenon is among the primary concerns of the healthcare system (Fernandez-Lazaro et al., 2019). Therefore, improving adherence to treatment regimens among cardiac patients is essential. A variety of interventions such as improving patients' education, using treatment reminders, cognitive behavioral interventions, reduction of medication costs, and engagement of healthcare team members is proposed to improve medical adherence (Simon et al., 2021). Alongside personal and social characteristics, healthcare team members including physicians and nurses, can also significantly influence adherence to treatment regimens (De Baetselier et al., 2022). Nurses are vital members of the healthcare team, spending considerable time caring for patients and assisting them with their daily tasks (Alghamdi et al., 2023). Nurses can improve medical adherence by providing knowledge, explaining the benefits and side effects of medication, and aiding patients in proper drug consumption (Konstantinou et al., 2020; Anderson et al., 2020).

Establishing effective and appropriate communication between nurses and patients can play a significant role in patient learning and acceptance of nurses' recommendations regarding treatment adherence regimens (Stolt et al., 2016). Trust is necessary to establish effective interpersonal relationships and is a substantial part of healthcare provision (Konstantinou et al., 2020; Anderson et al., 2020). Trust means the trustors believe that the trustees will apply the necessary interventions to meet the trustor's needs, expectations, and desires (Simon et al., 2021). Patient trust refers to the patient's expectation of optimal performance from the treatment staff including physicians, nurses, etc. (Hong et al., 2018).

Trust lies at the core of nurse-patient relationships and should exist in all clinical settings (Zhao et al., 2017). Trust in nurses enhances treatment satisfaction (Lindner, 2018), improves quality of care and reduces complaints against nurses (Çoşkun Palaz and Kayacan, 2023), increases patient compliance and cooperation with medical care, leads to improved healthcare delivery, and provides patients with a greater sense of security (Leslie and Lonneman, 2016). Conversely, the absence of trust in nurses can lead to the formation of dysfunctional cycles in nurse-patient relationships (Stolt et al., 2016) and may have undesirable psychological and therapeutic consequences for patients (Aghasi et al., 2021).

Healthcare providers have a significant role in enhancing medication adherence (Rashidi et al., 2020). Medication adherence can be influenced by mutual trust between patients and nurses. According to the literature it is estimated that there may be a relationship between patients' trust in nurses and adherence to treatment regimens (Aghasi et al., 2021). This study aimed to investigate the correlation between trust in nurses among cardiac patients and their medication adherence and explore the predictors of medication adherence.

## 2 Methodology

This descriptive correlational design was conducted among "302″ cardiac patients hospitalized at Amir Al-momenin Teaching Hospital affiliated with Maragheh University of Medical Sciences, northwest of Iran. The center covers a wide spectrum of patients from neighboring cities and serves as a referral center for cardiovascular patients in the southern regions of East Azerbaijan and northern regions of West Azerbaijan provinces.

### 2.1 Participants

All hospitalized patients in cardiac departments, including the cardiac internal ward, Coronary Care Unit (CCU), post-CCU ward, and post-angiography ward, were considered as the accessible population for the study. Inclusion criteria for participation in the study included hospitalization due to cardiac disease, having a history of taking heart medication for at least six months, being over 18 years old, being conscious, capable of verbal communication, being mentally stable, being hospitalized for more than two days, and providing informed consent to participation. Also, Patients who had special education related to medical sciences were excluded from the studies.

### 2.2 Sample size and sampling method

The sample size was calculated using the sample size formula to estimate the mean, based on findings from a similar previous study (Hood et al., 2018), considering an alpha of 0.05. The final sample size was calculated at least 325 eligible participants. In this study, participants were selected using a stratified sampling method. The eligible participants were selected randomly based on the share of each hospital department in the monthly hospitalizations of cardiac patients. Departments with more hospitalizations were allocated a larger share of the study sample.

$$n=\frac{{z^{2}}_{1-\alpha /2}\;*\;S^{2}}{d^{2}}=\frac{\left(1.96^{2}*23^{2}\right)}{{\left(2.5\right)}^{2}}=325$$

### 2.3 Data collection

Data was collected after the study proposal was approved by the Research Council and ethical committee of Maragheh University of Medical Sciences. The principal investigator visited the study setting and provided the study questionnaires to the participants after obtaining the necessary permissions and participants' consent. For participants unable to complete the questionnaires for any reason, preferably, the questionnaires were completed by the patient's companion, and if unavailable, by the researcher themselves. In this study, the demographic characteristics of participants were measured using a socio-demographic questionnaire, patients' trust in nurses was measured using the Trust in Nurses Scale, and patients’ treatment adherence was measured using the Medication Adherence Scale. Data was gathered from October 2023 to January 2024.

### 2.4 Socio-demographic questionnaire

This questionnaire was designed by the researchers based on personal experiences and a review of texts related to variables influencing medication adherence. Socio-demographic characteristics such as age, gender, education level, marital status, economic status, medical history, and hospitalization history were included. After designing the questionnaire, it was reviewed and approved for validity by 10 faculty members.

### 2.5 Trust in Nurses Scale (TNS)

This scale is a standardized tool designed by Cabral and Radwin in 2010 (Radwin and Cabral, 2010). In this study, the Persian version of the tool was used (Nooripour et al., 2015). TNS consists of five items scored on a 5-point Likert scale from 0 to 5 (never - always). The minimum score on this questionnaire is zero, and the maximum score is 25. A higher score indicates greater patient trust in the nurse. Cabral and Radwin in their study report that the TNS is a valid scale in terms of content and construct validity. Also, they reported an acceptable consistency reliability for the TNS. (α= 0.77) (Radwin and Cabral, 2010). Stolt et al. in a cross-cultural cross-sectional study reported an excellent structural validity for the TNS that The KMO(Kaiser-Meyer-Olkin) value ranged from 0.905 to 0.925. Also, Cronbach's alpha coefficient ranged from 0.78 to 0.95 for the TNS (Lindner, 2018). The Persian version of the tool has also been evaluated in several domestic studies and has been reported to have satisfactory validity and reliability (Nooripour et al., 2015; Ahmadpour et al., 2020). In this regard, Nooripour et al. reported that the TNS has an acceptable validity and is consistent with the Iranian context. They reported a Cronbach's alpha of 0.84 for TNS. (Nooripour et al., 2015)^,^. In the present study, the tool was re-evaluated to ensure the reliability of the questionnaire, and a Cronbach's alpha coefficient of 0.91 was calculated.

### 2.6 Medication adherence scale

This scale is a standardized tool designed by Madanlou and colleagues as part of a master thesis (Modanloo, 2013). The scale consists of 40 items categorized into 7 domains: attention to treatment (9 items), willingness to participate in treatment (7 items), ability to adhere (7 items), integration of treatment into life (5 items), medication adherence (4 items), commitment to treatment (5 items), and doubts about treatment implementation (3 items). Scoring is based on a 5-point Likert scale ranging from 0 to 5 (completely - not at all). The minimum score is zero, and the maximum score is 200. A higher score indicates better therapeutic adherence. According to the scoring of the scale: scores of 150–200 indicate very good medication adherence, 100–149 indicate good medication adherence, 50–99 indicate moderate medication adherence, and 0–49 indicate poor medication adherence. Seyed fatemi et al. declared that the medical adherence scale is valid and is suitable for use in the Iranian context. They reported a Cronbach's alpha of 0.92 for the scale (Seyed Fatemi et al., 2018). In the present study, Cronbach's alpha coefficient for the questionnaire was calculated to be 0.86.

### 2.7 Data analysis

Study data was analyzed using SPSS software (IBM SPSS statistics, version 21, IBM Corp, Armonk, NY, USA). Descriptive statistics including frequency, percentage, mean, standard deviation, median and first and third quartiles were used in descriptive tables for data analysis. Kolmogorov-Smirnov test was employed to assess the normality of quantitative variables. Due to the non-normality of quantitative variables, non-parametric tests such as Kruskal-Wallis was used to compare quantitative variables with qualitative variables of more than two groups and Mann-Whitney was used for qualitative variables of only two groups. Spearman correlation coefficient were used to analyze the relationships between medication adherence and quantitative variables. Additionally, quartile regression analysis was used to explore further associations and to detect the variables predict the medication adherence. All variables with a p-value <0.2 in univariate analysis were included in the model. Finally, variables with p-values <0.05 in quartile regression were introduced as predictive variables for medication adherence in the model.

### 2.8 Ethical approval

The study was approved by the Research Council and the Ethics Committee of Maragheh University of Medical Sciences (Ethics Code: IR.MARAGHEHPHC.REC.1402.017) on 7 June 2023. Also, the research conforms to the provision of the Declaration of Helsinki in 1995 (Goodyear et al., 2007). Before collecting the data, the participants were provided with information about the research and its objective and each participant completed a written informed consent prior to participation. They were also assured about the confidentiality of their information, and their right to withdraw the study at any stage.

## 3 Results

### 3.1 Demographic characteristics of participants

In this study, the data of 302 participants was analyzed. Analysis revealed that the mean age of participants was 63±13 years, and 172 patients (56.7 %) were male. It was found that most of the participant were economically in moderate level (67.2 %) and 47 % of them were hospitalized due to coronary artery disease (CAD). Further information regarding the socio-demographic characteristics of participants is presented in Table 1.

**Table 1:** Demographic characteristics and the Mean (SD) of medication adherence and patients trust in nurses.

Variables		Number(%)
Gender	Male	174 (57/6)
	Female	128 (42/4)
Educational level	Illiterate	61(20.2)
	Primary and middle school	183(60.6)
	High school	38(12.6)
	Bachelor	20 (6.6)
Economic status	Low	99(32.8)
	Medium	203(67.2)
	High	0(0 %)
Present disease	CAD	142(47.0)
	Hypertension	86(28.5)
	Other	74(25.5)
Inpatient ward	Ccu	131(43.3)
	Post CCU	140(46.4)
	Post angiography	31(10.3)
Age(*M*±SD)	63±13	
Duration of disease(*M*±SD)	2 ± 3 (1–20 years)	
Length of hospitalization(*M*±SD)	6 ± 11 (2–60 days)	
Medication adherence(*M*±SD)	91.6 ± 6.9	
Trust in nurses (*M*±SD)	10.5 ± 3.9	

### 3.2 Trust in nurses, medication adherence, and related demographic characteristics

Results showed that the mean score of medication adherence and trust in nurses among participants was 91.6 ± 6.9 and 10.5 ± 3.9, respectively. The results indicated significant differences in the median of medication adherence variable among different groups based on the type of disease, with the highest adherence among patients with coronary artery disease and the lowest among patients with hypertension. More details are provided in Table 2.

**Table 2:** Comparison of medication adherence between the qualitative variables.

Variable		Medication adherence	
		Median (First and Third Quartiles)	P-Value
Gender	Male	91.5(64,117)	0.413^a^
	Female	85(68,104)	
Educaional level	Illiterate	81(70, 104)	0.504^b^
	Primary and middle school	93(62, 121)	
	High school	95.5(68, 114)	
	Bachelor	80(79, 101)	
Economic status	Low	93(70, 108)	0.124^a^
	Medium	91(64,109)	
Present disease	Coronary artery disease	103(80, 117)	<0.0001^b^
	Hypertension	64(54, 93)	
	Other	81(70, 108)	
Inpatient ward	CCU	79(62,121)	0.122^a^
	Post CCU and Post Angiography	93(77,104)	

a Mann–Whitney U test.

b PKruskal–Wallis test.

### 3.3 Predictors of medication adherence

Analysis revealed a significant statistical relationship between medication adherence and patient trust in nurses. Also, a positive correlation was found between medication adherence and length of hospitalization. More details are presented in Table 3. In this study, a quartile regression analysis model was used to investigate the predictors of medication adherence. In this regard, economic status, present disease, trust in nurses, length of hospitalization, and inpatient ward were entered into the initial model. After removing non-significant variables, only patients’ trust in nurses and present disease remained in the final model, with an R-squared of 20 %. Further details are provided in Table 4.

**Table 3:** Correlation of medication adherence with quantitative variables.

variables	rs^a^	P-value
Age	0.04	0.513
Duration of disease	−0.06	0.337
Length of hospitalization	0.35	0.015
Trust in nurses	0.61	<0.0001

a Spearman correlation coefficient.

**Table 4:** Quartile regression model for predicting medication adherence.

variable	Coefficient	95 % conf. Interval for Coefficient		P-value
Trust in nurses	2.625	1.401423	3.848577	<0.0001
Present disease	14	8.429208	19.57079	<0.0001

## 4 Discussion

This descriptive-correlational study aimed to investigate the correlation between trust in nurses among cardiac patients and their medication adherence and explore the predictors of medication adherence in a referral center for cardiac care in northwest Iran. Findings indicated that patients' medication adherence was at a moderate level and their trust in nurses was less than average. A positive relationship between medication adherence and patients' trust in nurses was detected.

This study showed that medication adherence among cardiac patients is moderate. It has been found that medication adherence was in a moderate level in some similar previous studies (Zirak et al., 2020)^,^. A previous study reported moderate medication adherence among elderly patients with atrial fibrillation (Houshyar et al., 2021). Zirak et al. (2020) revealed that medication adherence among patients undergoing coronary artery angioplasty was moderate (Zirak et al., 2020). Zyryanov et al. reported that medication non-adherence is about 50 % among coronary artery patients (Zyryanov et al., 2020). Mashouf Rad et al. reported that about 78.75 % of cardiovascular patients had moderate adherence to their medication regimen (Mashouf Rad et al., 2022). Finding of the present study indicated that the medication adherence status was suboptimal among cardiac patients; which may pose multiple challenges for patients, their families, healthcare systems, and insurance institutions. Existing evidence shows that poorer adherence to medications predicts poorer prognoses of the cardiac disease (Chen et al., 2022). So, Medication adherence among cardiac patients is of paramount importance. Therefore, necessary measures should be taken to improve medication adherence in cardiac patients.

A worrying finding was that the cardiac patient's trust in nurses was less than average which contradicts several previous studies. Ahmadpour et al. reported that hemodialysis patients had a high level of trust in their nurses (Ahmadpour et al., 2020). Another study revealed a high level of trust in nurses among oncology patients (Ozaras and Abaan, 2018). Bahari et al. reported that emergency department patients had a high level of trust in nurses. (Bahari et al., 2024). Also, in another previous study, It was determined that the level of trust in nurses of patients receiving COVID-19 treatment was high (Çoşkun Palaz and Kayacan, 2023). The present finding requires further investigation. The reasons for cardiac patients' low trust in nurses should be explored, and necessary measures should be applied to remove obstacles and improve patients' trust in nurses.

One of this study's interesting and important finding was that patients' trust in nurses positively predicted medication adherence among cardiovascular patients. A similar study also revealed that greater patient trust in nurses leads to increased medication adherence (Ahmadpour et al., 2020). In another study, patients' trust in care providers was identified as an important predictor of medication adherence (Kvarnström et al., 2021). Results of the presents study indicated that an increase in patients trust in nurse significantly improved the medication adherence. Based on the results, improving patients trust in nurses is a key factor regarding improving of medication adherence. Nurses can play an important role in enhancing medication adherence among cardiac patients (Rashidi et al., 2020). Nurses, as the largest group providing healthcare and educational services, have the most contact with patients throughout the day (Rashidi et al., 2020). Trust in nurses instills a sense of security in patients and enhances their compliance and cooperation with their care and treatment measures (Leslie and Lonneman, 2016). Therefore, appropriate measures should be taken to enhance patients' trust in nurses. Establishing a professional relationship between patients and nurses leads to increased trust in nurses (Afriyie, 2020; Allande-Cussó et al., 2022). So, employing nurses with high communication skills and trying to improve their communication skills is proposed (Aghasi et al., 2021). Also, improving nurses' competence and adherence to ethical codes will guarantee the formation of patients' trust in nurses (Ozaras and Abaan, 2018). On the other hand, increasing patient-nurse interaction by reducing the nurse-patient ratio and reducing or eliminating non-clinical tasks can provide the basis for increasing patients' trust in nurses (Rashidi et al., 2020; Morady et al., 2023).

In this study, it was found that medication adherence varied significantly based on the type of cardiovascular disease, with the highest adherence among patients with coronary artery disease and the lowest among patients with hypertension. Similarly, a study conducted on individuals with high blood pressure in Spain also showed low treatment adherence among them (Lor et al., 2019). In contrast, high adherence to treatment was reported in patients undergoing angiography (Kähkönen et al., 2018). The study findings indicated low treatment adherence among patients with high blood pressure compared to patients with coronary artery disease. So the study findings highlight the importance of addressing treatment adherence in patients with high blood pressure along with other cardiac diseases (Burnier and Egan, 2019). In the present study patients' trust in nurses and the type of cardiac disease were identified as the main predictors of medication adherence. Considering the type of disease as an unmodifiable variable, trust in nurses is the main modifiable factor that plays an important role in increasing medication adherence of cardiovascular patients.

## 5 Conclusion

The findings of the present study indicated that the level of medication adherence among patients with cardiovascular disease was moderate. The study findings also showed a direct relationship between patients' trust in nurses and medication adherence, suggesting that enhancing patients' trust in nurses can be the key to improving medication adherence among cardiac patients. Therefore, based on the findings, it is recommended that necessary measures be taken to enhance patients' trust in nurses by providing communication skills training and attracting interested individuals with high communication skills, along with other proposed solutions to improve patients' trust in nurses. On the other hand, given the low medication adherence among patients with hypertension compared to other cardiovascular patients and the high importance of hypertension due to its complications and high mortality, priority should be given to increasing medication adherence in these patients.

### 5.1 Research limitations and recommendations for future studies

The data of the present study were collected using some self-reporting scales. Therefore, the study results may not fully reflect all the realities in this area. However, efforts have been made to collect accurate data by continuously being present in the study environment and gaining patients' trust. It is recommended that further studies be conducted using different methodologies, including experimental studies, to further explore the relationship between patient trust in nurses and medication adherence. Another limitation of this study is the lack of data collection related to trust in physicians and other service providers and their impact on medication adherence among cardiovascular patients, which suggests the need for further studies. Also, it was found that the patients trust in nurse was less that average that need further investigation.

### 5.2 Ethics approval and consent to participate

The study was approved by the Research Council and the Ethics Committee of Maragheh University of Medical Sciences (Ethics Code: IR.MARAGHEHPHC.REC.1402.017) on 7 June 2023. Also, the research conforms to the provision of the Declaration of Helsinki in 1995. Before collecting the data, the participants were provided with information about the research and its objective and each participant completed a written informed consent prior to participation. They were also assured about the confidentiality of their information, and their right to withdraw the study at any

## Ethics code

IR.MARAGHEHPHC.REC.1402.017.

## Funding

This study was supported by the Maragheh University of Medical Sciences, deputy of research.

## CRediT authorship contribution statement

**Ebrahim Aliafsari Mamaghani:** Writing – review & editing, Writing – original draft, Validation, Supervision, Methodology, Conceptualization. **Ali Soleimani:** Writing – review & editing, Writing – original draft, Methodology, Formal analysis, Data curation, Conceptualization. **Mohammad Zirak:** Writing – review & editing, Writing – original draft, Validation, Supervision, Methodology, Data curation, Conceptualization.

## Declaration of competing interest

The authors declare that they have no known competing financial interests or personal relationships that could have appeared to influence the work reported in this paper.
